# Development of a PMGDNI model to predict the probability of three-month unfavorable outcome acute ischemic stroke after endovascular treatment: a cohort study

**DOI:** 10.1186/s12883-024-03960-1

**Published:** 2024-12-05

**Authors:** Chao Yang, Jingying Wang, Ruihai Zhang, Yiyao Lu, Wei Hu, Peng Yang, Yiqing Jiang, Weijun Hong, Renfei Shan, Yinghe Xu, Yongpo Jiang

**Affiliations:** 1 Department of Emergency Medicine, Taizhou Hospital of Zhejiang Province Affiliated to Wenzhou Medical University, Taizhou, China; 2 Department of Neurology, Taizhou Hospital of Zhejiang Province Affiliated to Wenzhou Medical University, Taizhou, China; 3 Department of Neurosurgery, Taizhou Hospital of Zhejiang Province Affiliated to Wenzhou Medical University, Taizhou, China; 4 Department of Critical Care Medicine and Emergency Medicine, Taizhou Hospital of Zhejiang Province Affiliated with Wenzhou Medical University, No.150, XiMen Street, Taizhou, China

**Keywords:** Large vessel occlusion, Endovascular treatment, Mechanical thrombectomy, Predictive model, Functional independence

## Abstract

**Background:**

Patients with acute large vessel occlusion stroke (ALVOS) may exhibit considerable variability in clinical outcomes following mechanical thrombectomy (MT). This study aimed to develop a novel statistical model predicting functional independence three months post-endovascular treatment for acute stroke and validate its performance within the cohort.

**Method:**

Consecutive patients undergoing endovascular treatment for acute stroke with large vessel occlusion were randomly divided into a modeling group and a validation group in a 7:3 ratio. Independent risk factors were identified through LASSO regression and multivariate logistic regression analyses, leading to the development of a prognostic model whose performance was rigorously validated.

**Results:**

A total of 913 patients were screened, with 893 cases included. The modeling group comprised 625 cases, and the validation group included 268 cases. Identified independent factors for adverse outcomes after endovascular treatment of acute ischemic stroke (AIS) were pneumonia (OR = 4.517, 95% CI = 2.916–7.101, *P* < 0.001), mechanical ventilation (OR = 2.449, 95% CI = 1.475–5.148, *P* = 0.001), admission GCS ≥ 8 (OR = 0.365, 95% CI = 0.167–0.745, *P* = 0.008), dysphagia (OR = 2.074, 95% CI = 1.375–3.126, *P* < 0.001), and 72-hour highest Na ≥ 145 (OR = 2.794, 95% CI = 1.508–5.439, *P* = 0.002), along with intracranial hemorrhage (OR = 2.453, 95% CI = 1.408–4.396, *P* = 0.002). These factors were illustrated in a PMGDNI column chart. The area under the ROC curve for the modeling group was 82.5% (95% CI = 0.793–0.857), and for the validation group, it was 83.7% (95% CI = 0.789–0.885). The Hosmer-Lemeshow test indicates that there is no statistically significant difference (*P* > 0.05) between the predicted and actual probabilities of adverse outcomes. The clinical decision curve demonstrated optimal net benefits at thresholds of 0.30-1.00 and 0.25-1.00 for both training and validation sets, indicating effective clinical efficacy within these probability ranges.

**Conclusion:**

We have successfully developed a new predictive model enhancing the accuracy of prognostic assessments for acute ischemic stroke following EVT. It provides an individual, visual, and precise prediction of the risk probability of a 90-day unfavorable outcome.

**Supplementary Information:**

The online version contains supplementary material available at 10.1186/s12883-024-03960-1.

## Introduction

Acute ischemic stroke (AIS) stands as the leading cause of death and functional disability worldwide [1, 2]. More than 45.5% of deaths from stroke, and 71.7% of living ability lost because of stroke were in people younger than 75 years [2]. However, with the continuous development of clinical medicine, significant improvements have been made, especially through methods such as thrombolysis and endovascular thrombectomy, leading to a noticeable decrease in death and disability rates [3]. Nevertheless, for stroke patients with large vessel occlusion (LVO), long-term disability still affects approximately 50% of individuals [4]. Currently, endovascular therapy (EVT) has been established as an effective method for treating acute large vessel occlusion stroke (ALVOS) [5, 6], with efficient recanalization of occluded vessels being a key benefit for ALVOS patients. However, in clinical practice, there remain patients whose clinical outcomes cannot be improved even after successful recanalization of occluded vessels and restoration of blood flow [7, 8]. Therefore, the development of a feasible LVO EVT result prediction model has become a top priority.

Previous studies have identified multiple predictors of key clinical outcomes after stroke, including complications, clinical examination results, brain edema, reperfusion injury, and high scores on the NIHSS scale [9–12]. Based on these factors, numerous prediction models have been developed to predict the prognosis of individual patients receiving EVT treatment. However, due to the small sample size and complex data collection of these models, and the limited inclusion of factors such as anterior circulation occlusion, their practical performance is suboptimal, and they fail to address the complexity of various parameters that may influence the clinical outcomes of AIS endovascular treatment. As a result, no model has emerged as the preferred choice for EVT patients [9, 13].

With the increasing prevalence of machine learning in recent years, the utilization of machine learning models in clinical data analysis has become more common. However, the presence of biased machine-learning models can potentially lead to a reduction in accuracy [14]. While machine learning offers significant benefits, it is also important to acknowledge its limitations. It may not always outperform traditional techniques in all scenarios [15]. Therefore, it is essential to recognize the strengths and weaknesses of both machine learning and traditional models and utilize them in a more balanced and judicious manner for clinical data applications.

As such, in pursuit of aiding treatment strategies and clinical decision-making, our research focuses on patients with acute large vessel occlusion stroke, aiming to explore the risk factors that may impact the prognosis of these patients after endovascular treatment, and construct a predictive model. Clinicians can utilize predictive models to prioritize patients at high risk and administer early intervention and treatment, consequently diminishing the incidence of adverse outcomes.

## Methods

### Participants

**Fig. 1:**
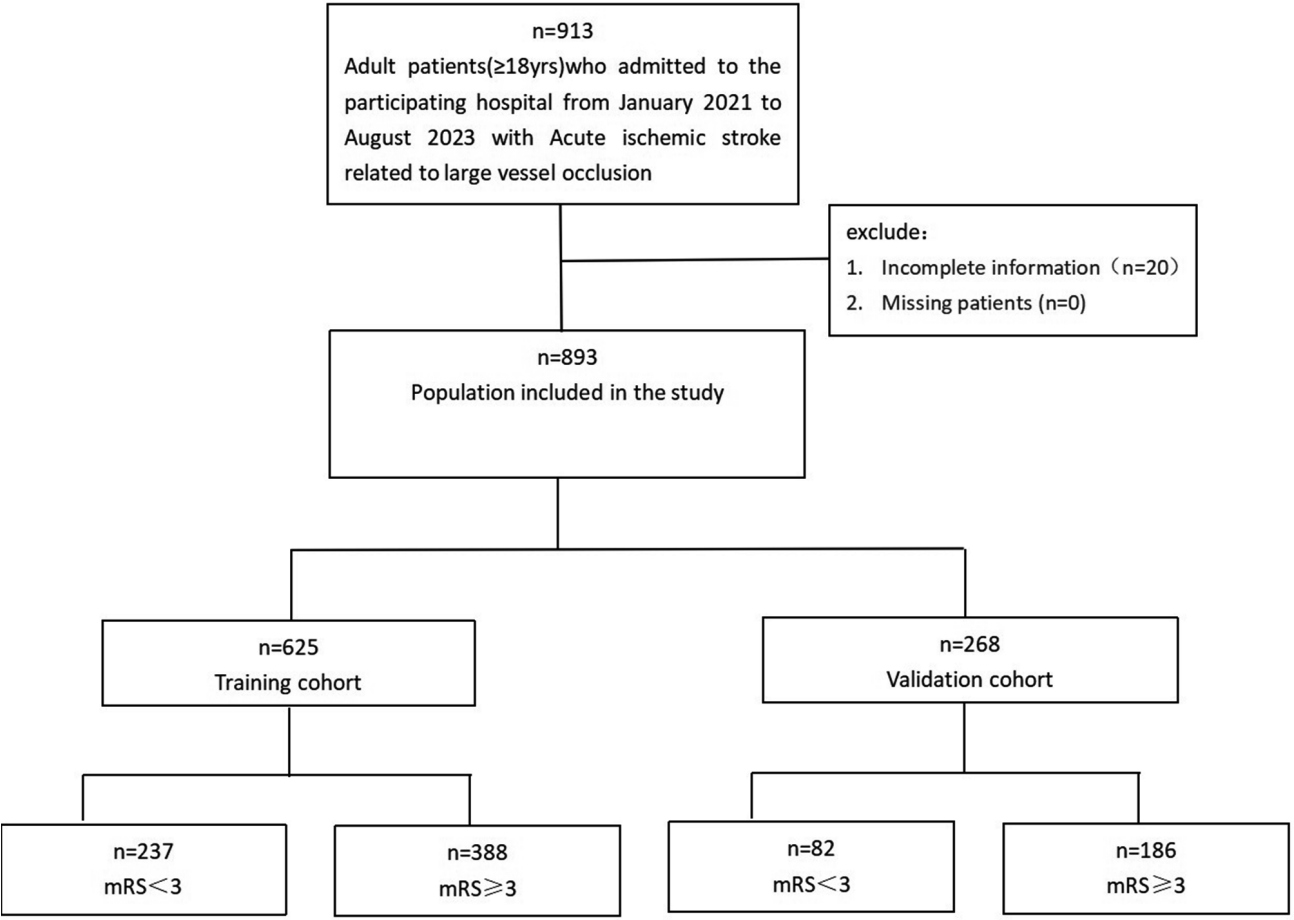
Study flow diagram

From January 2021 to August 2023, consecutive acute stroke patients at three hospitals of Taizhou Enze Medical Center (Group) were retrospectively included in this study. A total of 913 patients were initially chosen, but after applying inclusion and exclusion criteria, 893 patients were ultimately included. The patients were randomly allocated in a 7:3 ratio, with 625 patients being assigned to the training module group and 268 patients to the validation group. The patients were categorized into a good prognosis group (mRS < 3) and a poor prognosis group (mRS ≥ 3) based on their functional independence [16], as illustrated in Fig. 1. The modified Rankin Scale (mRS) score was obtained after 90 days of onset through telephone and outpatient follow-up from experienced neurologists. This study was approved by the Ethics Committee of Enze Hospital, Taizhou Enze Medical Center (Group) (K20221104), and was conducted by following the Helsinki Declaration and the law of China. The inclusion criteria required patients to be 18 years old or above, diagnosed with acute cerebral infarction confirmed via CTP or CT, and have large vessel occlusion necessitating endovascular treatment. Exclusion criteria included patients who had not consented to endovascular treatment, did not fulfill the criteria for endovascular treatment, had incomplete medical record information, or had an MRS score > 3 points before admission.

### Study procedure

Collect clinical data of cases through the hospital medical record system, including the following information:1.General information: age, gender, onset time, admission temperature, admission blood pressure, underlying diseases, etc.2. Clinical data: (a) Laboratory examination: White blood cell count, lymphocyte count, neutrophil count, platelets, hemoglobin, CRP, PCT, triglycerides, creatinine, blood glucose, coagulation function, international standardized ratio, activated partial prothrombin time, fibrinogen, thrombin time, D-dimer, albumin, etc. upon admission and after 72 h. Electrolyte changes were rechecked. (b) Imaging examination: blockage of blood vessels, cerebral hemorrhage, etc.3. Intervention and scoring: admission Glasgow Coma Scale (GCS) score [17], National Institutes of Health Stroke Scale (NIHSS) score, 90-day mRS score, pneumonia, mechanical ventilation, swallowing dysfunction, drug thrombolysis, etc. Please note that the above information is collected through the hospital’s medical record system.

### Statistical method

Statistical analysis was performed using SPSS 27.0 and R language (4.3.2) was utilized for data analysis. The two-tailed p-value < 0.05 is considered to indicate a statistically significant difference. χ² tests, t-tests, or Mann-Whitney U-tests were used to compare the differences between baseline characteristics. The optimal threshold for predicting clinical prognosis scores was determined using the Youden index, which maximizes sensitivity and specificity. LASSO regression was performed using the “glmnet” package, utilizing 10-fold cross-validation and the lambda 1se criterion to select the optimal factors influencing the outcome. Subsequently, multiple forward stepwise logistic regression was conducted on the selected factors from the LASSO regression using the “glm” package [18]. Using the “rms” package in R, nomograms and forest plots were constructed. The 95% confidence intervals (CI) were estimated through 1,000 bootstrap samples. The sample was randomly divided into model and validation groups in a 7:3 ratio. The discriminative ability of the model and validation cohorts was evaluated by plotting the Area Under the Receiver Operating Characteristic (ROC) Curve (AUC) using the “fbroc” and “rms” packages in R. Calibration tests were conducted on both the model and validation groups using the Hosmer-Lemeshow test, estimating observed vs. predicted rates and computing the P-value for goodness-of-fit. Decision Curve Analysis (DCA) was used to quantify net benefits across varying threshold probabilities by plotting the DCA for both groups with the “rmda” package. Finally, the differences between our model, the NAC model, the COACHS model, the NADE model, and the START model were compared through an analysis of the ROC curves.

## Results

The final cohort consisted of 893 patients with a median age of 70 years (interquartile range: 60–77 years). Among them, 61.5% were male and 38.5% were female. The training set included 625 cases, while the validation set included 268 cases (Table 1).

**Table 1:** Baseline characteristics of all patients in the training cohort and validation cohort

Variables	Total	Training cohort	Validation cohort	*P*-value
	*n* = 893	*n* = 625	*n* = 268	
Age (years)	70(60,77)	70(61,77)	68(59,77)	0.041
Gender				
Male	549(61.5)	384(61.4)	165(61.6)	0.971
Female	344(38.5)	241(38.6)	103(38.4)	
Admission situation				
time of onset(hour)	5(3,8)	5(3,8)	5(2.5,9)	0.847
temperature(℃)	36.6(36.5,36.8)	36.6(36.5,36.8)	36.6(36.5,36.8)	0.594
systolic pressure(mmHg)	150(143,167)	150(133,150)	150(135,168)	0.422
GCS	11(9, 12)	11(9, 12)	11(8, 12)	0.133
NIHSS	13(8, 18)	12(8, 18)	13(9, 19)	0.163
disease history				
Hypertension	545(61.0)	377(60.3)	168(62.7)	0.506
COPD	22(2.5)	15(4.0)	7(2.6)	0.851
cerebral infarction	167(18.7)	124(19.8)	43(16.0)	0.183
Atrial fibrillation	252(28.2)	183(29.3)	69(25.7)	0.282
heart failure	45(5.0)	31(5.0)	14(5.2)	0.869
myocardial infarction	14(1.6)	11(1.8)	3(1.1)	0.480
Preoperative Laboratory parameters				
WBC(10^9/L)	8.2(6.6,10.6)	8.2(6.6,10.5)	8.1(6.5,10.7)	0.729
Neut(10^9/L)	5.9(4.4,8.2)	5.95(4.4,8.2)	5.9(4.3,8.3)	0.555
HB(g/L)	136(124,148)	136(123,148)	137(125,147)	0.662
PLT(10^9/L)	204(166,248)	204(166,248)	204(166,245)	0.857
CL(mmol/L)	104.7(102.2,107,0)	104.8(102.3,107.0)	104.6(102.0,107.0)	0.460
Na(mmol/L)	138.6(136.7,140.4)	138.7(136.8,140.4)	138.4(136.7,140.5)	0.438
Postoperatively Laboratory parameters				
WBC(10^9/L)	8.9(7.1,10.9)	8.9(7.1,10.8)	8.9(7.2,11.0)	0.652
Neut(10^9/L)	7.1(5.4,9.2)	7.1(5.3,9.2)	7.2(5.6,9.2)	0.709
HB(g/L)	124(113,136)	124(112,135)	124(115,137)	0.192
PLT(10^9/L)	194(157,232)	193(155,231)	195(161,237)	0.446
72 h high CL(mmol/L)	110.0(107.6,113.0)	110.0(107.5,113.0)	110.0(107.6,113.0)	0.633
72 h high Na(mmol/L)	142.0(140.1,144.5)	142.0(140.2,144.4)	141.9(140.0,144.9)	0.764
temperature(°)	36.5(36.5,37.0)	36.5(36.5,37.0)	36.5(36.5,37.0)	0.395
complication				
ICH	175(19.6)	127(20.3)	48(17.9)	0.406
pneumonia	400(44.8)	275(44.0)	125(46.6)	0.467
dysphagia	487(54.5)	336(53.4)	151(56.3)	0.477
operate				
mechanical ventilation	275(30.8)	194(31.0)	81(30.2)	0.809

Values were presented as n (%), mean (SD), or median (interquartile range)

GCS Glasgow Coma Scale, NIHSS National Institutes Of Health Stroke Scale, WBC White Blood Cell Count, Neut Neutrophil Count, HB Hemoglobin Concentration, PLT Platelet Count, CL Chlorine Ion Level, Na Sodium ion level, COPD Chronic Obstructive Pulmonary Disease, ICH Intracranial Hemorrhage

**Fig. 2:**
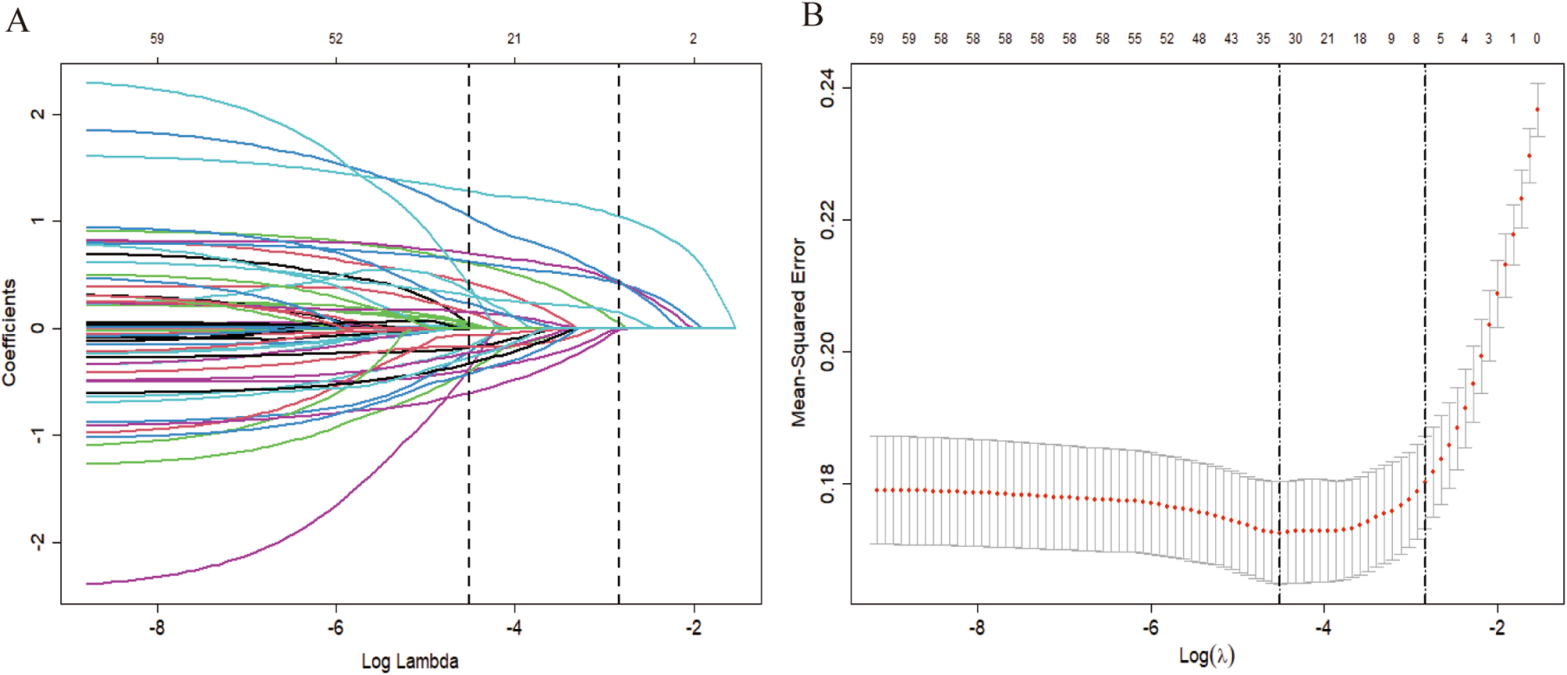
**A**: Clinical characteristics of LASSO coefficient spectrum. **B**: Generation of optimal penalty coefficient in LASSO through 10-fold cross-validation using λ. The value of λ is chosen based on the mean square error of the training set

**Fig. 3:**
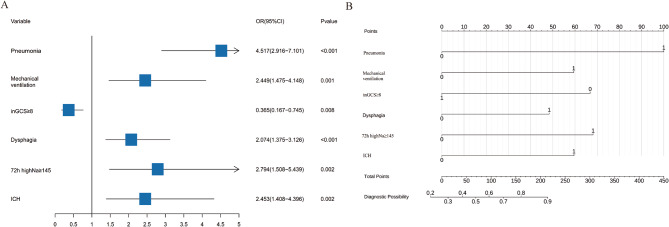
Column chart (**A**) and forest plot (**B**) of the PMGDNI model for predicting patient functional independence after 90 days

**Fig. 4:**
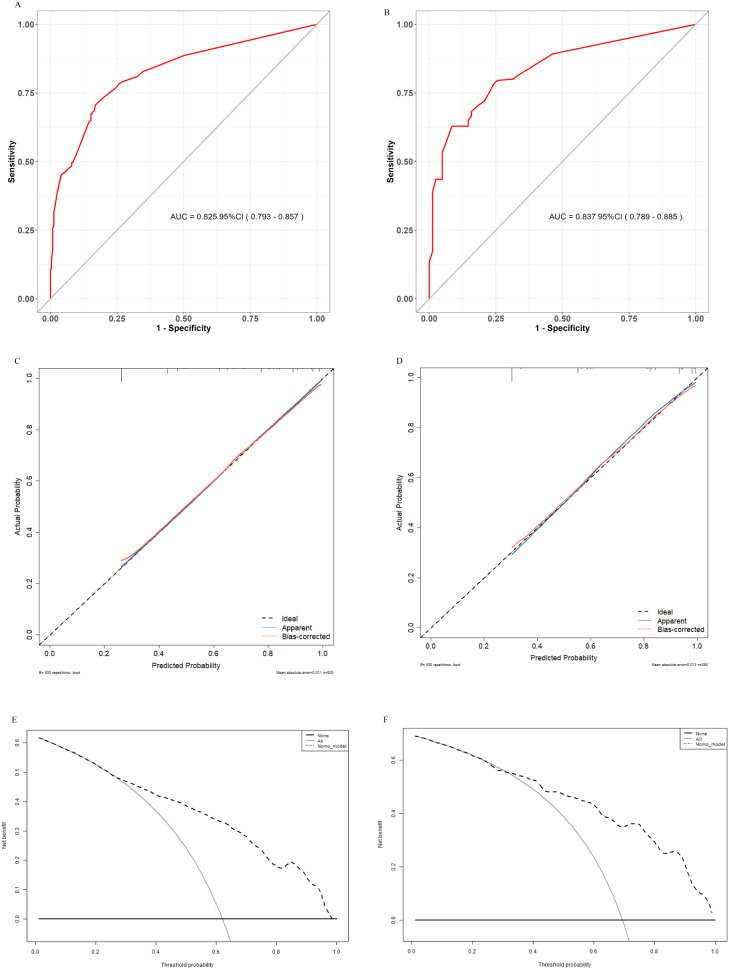
ROC curves and AUC values for training set (**A**) and validation set (**B**). (**C**) Calibration curves for the training dataset and (**D**) validation dataset. The black dashed line with a 45° angle represents ideal calibration, where the predicted probability equals the observed probability. Analysis of functional decision curves for (**E**) modeling group and (**F**) validation group predictions. The curves indicate that, when the probabilities range from 0.30 to 1.00 and from 0.25 to 1.00, the model predicts higher net benefits of functional strategies over default strategies after 90 days, with “all treatments” (all patients receiving positive intervention) and “no treatments” (no patients receiving positive intervention) showing higher net benefits

LASSO regression identified 28 influencing factors (Fig. 2), See Supplementary Fig. 1 for the regression system. Further analysis using multivariate logistic regression is presented below: pneumonia (OR = 4.517, 95% CI = 2.916–7.101, *p* < 0.001), mechanical ventilation (OR = 2.449, 95% CI = 1.475–5.148, *p* = 0.001), admission GCS ≥ 8 (OR = 0.365, 95% CI = 0.167–0.745, *p* = 0.008), dysphagia (OR = 2.074, 95% CI = 1.375–3.126, *p* < 0.001), and 72-hour highest sodium ≥ 145 (OR = 2.794, 95% CI = 1.508–5.439, *p* = 0.002), as well as intracranial hemorrhage (OR = 2.453, 95% CI = 1.408–4.396, *p* = 0.002). These factors were used to construct the final model, and the results were presented in the PMGDNI column and forest plots (Fig. 3). The sensitivity and specificity of the model were predicted using the ROC curve. The area under the curve was 82.5% (95% CI [0.793–0.857]) for the training set and 83.7% (95% CI [0.789–0.885]) for the validation set, indicating high consistency and excellent discrimination (Fig. 4).

The model’s discrimination and calibration can be utilized to predict the probability of adverse outcomes following endovascular treatment in acute ischemic stroke (Fig. 4). The Hosmer Lemeshow goodness-of-fit tests were conducted for the training set (X2 = 2.472, df = 6, p-value = 0.8716) and the validation set (X2 = 7.1721, df = 6, p-value = 0.3052), indicating good fit in both sets.

The DCA revealed that both the training and validation sets obtained maximum net benefits at thresholds ranging from 0.30 to 1.00 and 0.25 to 1.00, indicating good clinical efficacy within this probability range(Fig. 4).

**Fig. 5:**
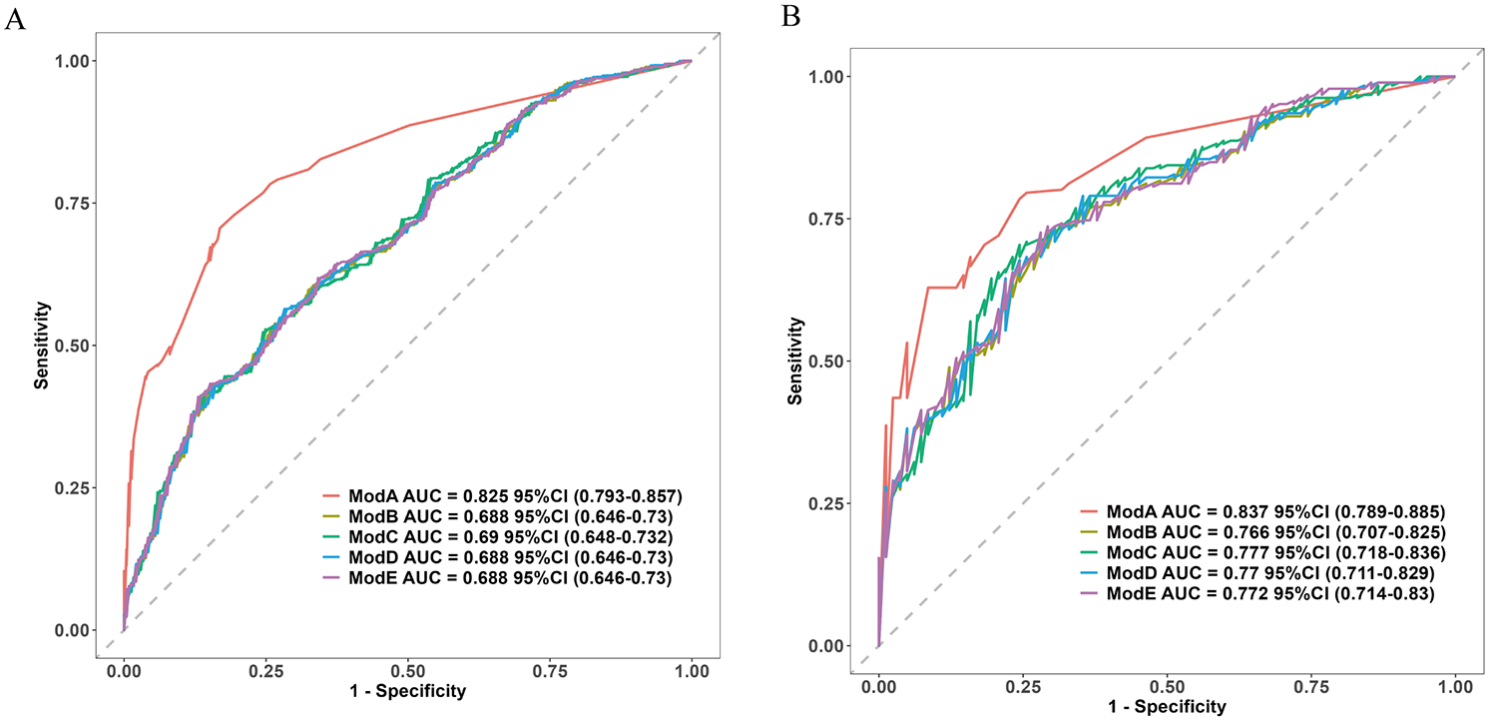
Comparison of Model (**A**) Modeling Group and (**B**) Validation Group with Other Models. The DeLong test revealed the following results: In the training set, model A compared to models B, C, D, and E all showed *P* < 0.01. In the validation set: the comparison of model A with model B yielded a P value of 0.01; model A versus model C, a P value of 0.02; model A versus model D, a P value of 0.03; and model A versus model E, a P value of 0.04

Additionally, we compared our models with previously reported ones such as the NAC model [19] [the training set AUCs of 0.688 (95% CI 0.646–0.730) and validation set 0.766 (95% CI 0.707–0.825)], COACHS model [20] [the training set AUCs of 0.690 (95% CI 0.648–0.732) and validation set 0.777 (95% CI 0.718–0.836)], NADE model [21] [the training set AUCs of 0.690 (95% CI 0.646–0.730) and validation set 0.770 (95% CI 0.711–0.829)], and START model [22] [the training set AUCs of 0.688 (95% CI 0.646–0.730) and validation set 0.772 (95% CI 0.714–0.830)] in Fig. 5, Our model demonstrated a higher area under the curve and more color differentiation, indicating superior performance in comparison to the aforementioned models.

## Discussion

We have constructed a prognosis model for stroke patients with large vessel occlusion through six variables. The six variables we propose are readily obtainable in clinical practice, allowing even primary hospitals to use this model to make simple predictions for stroke patients. In comparison with other models [19–21], we found that the PMGDNI model demonstrates superior predictive power.

In our study, we found that in stroke patients with large vessel occlusion, despite endovascular treatment and active intervention in some cases, only 35.7% of patients achieved good functional performance (mRS < 3) after 90 days. This is similar to a study conducted in 2015 where 34.3% of patients showed good functional performance [23], slightly better than the Netherlands’ multicenter collaboration which had a result of 32.6% [24], but much lower than the 46% achieved in the Goyal M meta-analysis [4]. In the meta-analysis, we found the rates of high functional independence outcomes were as follows: 32.6% in MR CLEAN [25], 53.0% in ESCAPE [26], 60% in SWIFT PRIME [27], 43.7% in REVASCAT [28], and 71% in EXTEND-IA [29]. The reason for these high rates can likely be attributed to the relatively small sample sizes in these studies and the inclusion of only patients who underwent thrombectomy within 8 h of symptom onset. Despite continued development and improvement of endovascular treatment techniques, there has been no significant improvement in the prognosis of large vessel occlusion in recent years. Furthermore, these findings highlight the importance of predicting the prognosis of stroke patients with large vessel occlusion.

### Pneumonia

We have analyzed several factors contributing to adverse outcomes through modeling and have found pneumonia to be the most influential factor. Currently, infection has been proven to be the primary cause of morbidity and mortality in patients with acute central nervous system injury [10]. Secondary immunodeficiency syndrome (CIDS) after a stroke significantly increases susceptibility to infection. Simultaneously, infection hinders the recovery of neurological function and increases both the incidence rate and mortality [10]. Pneumonia, the most common complication of ischemic stroke complicated with infection, shows a significant change in its incidence rate. Related literature reports that the incidence rate of stroke-related pneumonia (SAP) is 4.1–56.6% in the NICU and 17–50% in the MICU [30]. We observed a high incidence of pneumonia, which may be related to the presence of large vessel occlusion in the included population, as well as to the non-standard definition of post-stroke pneumonia.

### Mechanical ventilation and swallowing dysfunction

We have observed that patients with large vessel occlusion who require mechanical ventilation and experience swallowing dysfunction tend to have a worse prognosis, with incidence rates of adverse outcomes at about 88.0% and 80.5%, respectively. The incidence rates of pneumonia in these cases are 66.9% and 62.6%, although the statistical data available to us do not clarify the exact relationship between these time factors. However, it is not difficult to infer a strong link between pneumonia, mechanical ventilation, and swallowing dysfunction. Previous studies have also indicated that the incidence of pneumonia is connected to dysphagia and mechanical ventilation [31]. While there may be variations in how doctors evaluate the need for mechanical ventilation, a survey study has shown that the in-hospital mortality rate for stroke patients requiring mechanical ventilation can be as high as 52.7% [32]. Notably, our incidence of pneumonia is higher than the reported 40% incidence in stroke patients requiring mechanical ventilation in France [33]. Although swallowing dysfunction may increase the risk of reflux and aspiration, it is important to note that aspiration alone is not always sufficient to cause pneumonia, as around half of healthy adults may inhale during sleep without developing pneumonia [34]. Interestingly, previous research has indicated that pneumonia does not necessarily impact short-term mortality [31]; however, the presence of pneumonia, mechanical ventilation, and swallowing dysfunction may result in prolonged ICU hospitalization, leading to a poorer prognosis and higher mortality rates [33, 35].

### Intracranial hemorrhage

Intracranial hemorrhage (ICH) is a common and highly dangerous complication after surgery, occurring in approximately 40% of patients with anterior circulation occlusion of EVT [36]. It can result in adverse outcomes and increased mortality, ultimately reducing the risk-benefit ratio of endovascular treatment [37]. Although anterior circulation obstruction accounted for approximately 90.3% in our study, the incidence of bleeding in our cohort was much lower at 19.6%. Previous studies have shown a positive correlation between the number of times a stent thrombus retrieval device (SR) is passed and the increased risk of symptomatic intracerebral hemorrhage (SICH) [38, 39]. However, it is unclear whether such factors contribute to the differences in bleeding incidence. Nonetheless, there is mounting evidence that the occurrence of intracranial hemorrhage (ICH) or the possibility of early hemorrhagic transformation (HT) both significantly impacts the prognosis of these patients, leading to higher mortality and disability rates [40–42].

### Blood sodium

Blood sodium, as the most common electrolyte, plays a crucial role in maintaining bodily function. Hypernatremia was defined as two daily serum sodium values exceeding 145 mmol/l, which coincides precisely with the threshold used in our study. This condition was identified as an independent risk factor influencing the prognosis of endovascular treatment for large vessel occlusions. However, previous studies have indicated that hyponatremia is associated with a higher mortality rate and poorer clinical outcomes in stroke patients [43, 44]. There is limited research on high sodium levels, and hypertonic agents such as mannitol and hypertonic saline (HS) have been shown to reduce total brain water content and intracranial pressure, making them the primary drugs for treatment [45]. Hypertonic saline (HS) appears to act as a protective agent against brain damage. Although our cohort did not exclude patients using HS, this observation seems to contradict our results. Hypertonic saline is a complex treatment option for patients with elevated intracranial pressure levels. Its mechanism of action includes decreasing cell volume by extracting fluid from the brain and improving blood viscosity and rheology, leading to a decrease in brain blood volume, among other effects [46]. Several retrospective studies have suggested that hypernatremia is independently associated with an increased risk of death in patients with severe traumatic brain injury [47–49]. In this section of the study, the definition of high sodium values is very similar to our research. In animal experiments, it was found that hypernatremia caused by HS infusion after ischemia exacerbates the cortical infarct volume of transient focal cerebral ischemia [50]. However, we still cannot confirm a direct association between hypernatremia and brain damage.

### The glasgow coma scale (GCS)

The Glasgow Coma Scale (GCS) is the most widely used behavioral measure for assessing the severity of acute traumatic brain injury (TBI). A GCS total score of 8 is commonly used as the threshold to define “coma” for surgical purposes [51]。 Interestingly, we also identified 8 as the optimal cutoff value using the Youden index. Although there is currently no definitive GCS score threshold to differentiate stroke patient outcomes, the impact of GCS scores on stroke prognosis has been supported by numerous studies [52, 53]. Marielle K. in their study pointed out that a GCS score ≤ 8 is associated with a higher 30-day mortality rate among stroke patients [52].

### Limitations

Our study has several limitations. First, it is a retrospective analysis involving a limited number of patients, which necessitates prospective studies to validate the model’s performance. Second, we lack external validation to enhance the accuracy of our model. Third, for some patients, we obtained the mRS scores through telephone interviews, which may introduce bias in the scoring. Additionally, we only recorded HAP without differentiating between SPA and VPA. Although we included as much clinical data as possible, we cannot guarantee that there are no new potential independent factors that we may have overlooked.

## Conclusion

The model constructed using six independent risk factors—pneumonia, mechanical ventilation, admission GCS, swallowing difficulties, and intracranial hemorrhage—demonstrates a high predictive value for outcomes following endovascular treatment of acute ischemic stroke.

## Electronic supplementary material

Below is the link to the electronic supplementary material.

Supplementary Material 1: LASSO variables after model screening. Each line in the coefficient plot represents the retrospective coefficient of a specific feature, with the magnitude of the coefficient indicating the contribution of that feature to the model. (blood pressure: Postoperative blood pressure, 72 h High Na+: Maximum serum sodium level within 72 h post-admission, 72 h Low Cl: Minimum serum chloride level within 72 h post-admission, GCS: Glasgow Coma Scale score at admission, NIHSS: NIHSS score at admission).

## Data Availability

The data can be obtained from the corresponding author JYP (jyongpo8@163.com) upon reasonable request.

## Abbreviations

EVT: Endovascular Therapy
AIS: Acute Ischemic Stroke
LVO: Large Vessel Occlusion
ALVOS: Acute Large Vessel Occlusion Stroke
NIHSS: National Institutes Of Health Stroke Scale
mRS: Modified Rankin Scale
GCS: Glasgow Coma Scale
WBC: White Blood Cell Count
Neut: Neutrophil Count
HB: Hemoglobin Concentration
PLT: Platelet Count
CL: Chlorine Ion Level
COPD: Chronic Obstructive Pulmonary Disease
ICH: Intracranial Hemorrhage
