# Supplementary figures and images for: Development of a PMGDNI model to predict the probability of three-month unfavorable outcome acute ischemic stroke after endovascular treatment: a cohort study
Source: BMC Neurol. 2024 Dec 5;24:472. doi: 10.1186/s12883-024-03960-1 (PMC11619606; doi:10.1186/s12883-024-03960-1)

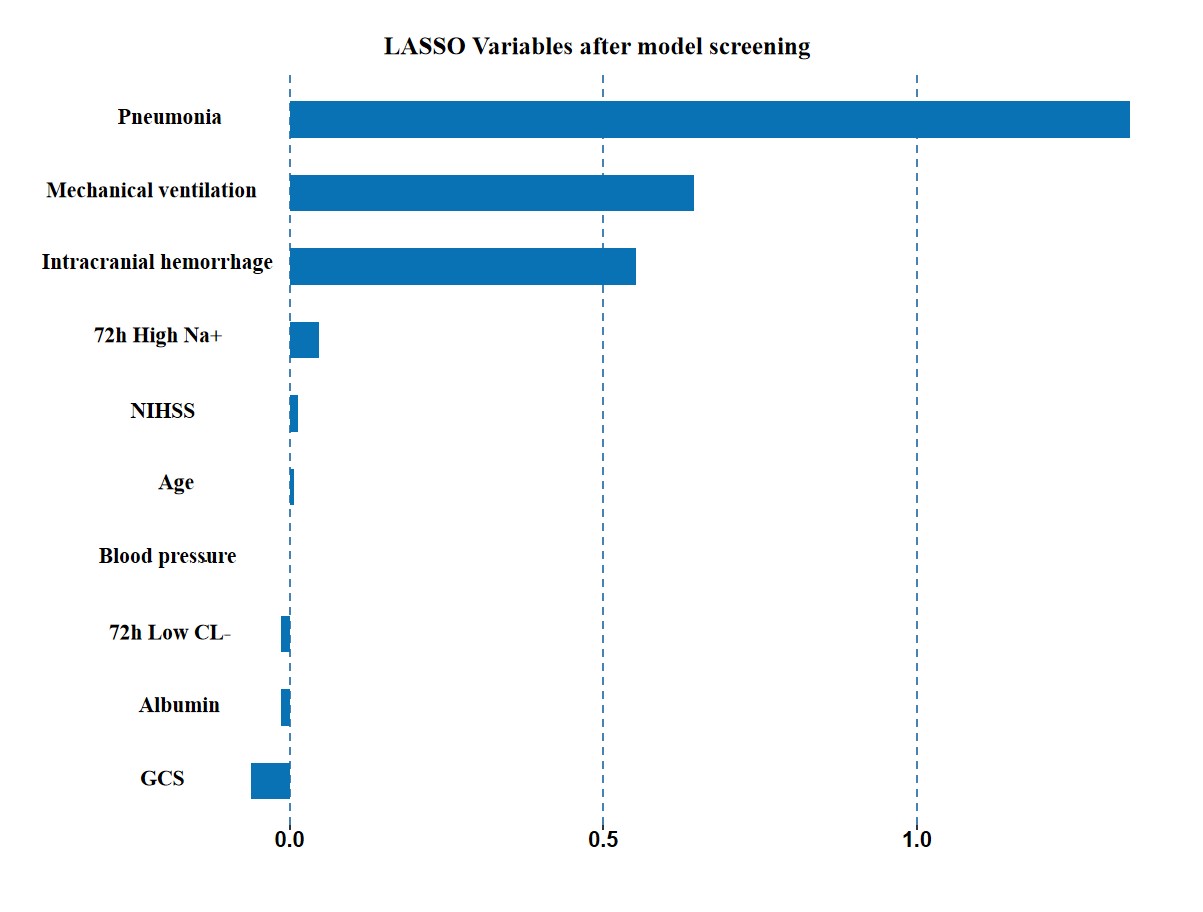

Supplement: Supplementary file 1 — Supplementary Material 1: LASSO variables after model screening. Each line in the coefficient plot represents the retrospective coefficient of a specific feature, with the magnitude of the coefficient indicating the contribution of that feature to the model. (blood pressure: Postoperative blood pressure, 72 h High Na+: Maximum serum sodium level within 72 h post-admission, 72 h Low Cl: Minimum serum chloride level within 72 h post-admission, GCS: Glasgow Coma Scale score at admission, NIHSS: NIHSS score at admission). [file 12883_2024_3960_MOESM1_ESM.tif]
